# Thyroid function and age-related macular degeneration: a prospective population-based cohort study - *the Rotterdam Study*

**DOI:** 10.1186/s12916-015-0329-0

**Published:** 2015-04-23

**Authors:** Layal Chaker, Gabriëlle HS Buitendijk, Abbas Dehghan, Marco Medici, Albert Hofman, Johannes R Vingerling, Oscar H Franco, Caroline CW Klaver, Robin P Peeters

**Affiliations:** Rotterdam Thyroid Center, Erasmus University Medical Center, Rotterdam, The Netherlands; Department of Internal Medicine, Erasmus University Medical Center, Rotterdam, The Netherlands; Department of Epidemiology, Erasmus University Medical Center, Rotterdam, The Netherlands; Department of Ophthalmology, Erasmus University Medical Center, Rotterdam, The Netherlands; Department of Internal Medicine, Rotterdam Thyroid Center, Erasmus University Medical Center, Endocrinology, Erasmus University Medical Center Rotterdam, Room Ee502, PO Box 2040, 3000 CA Rotterdam, The Netherlands

**Keywords:** Thyroid hormone, Thyroid function, AMD, Age-related macular degeneration

## Abstract

**Background:**

In animal models, lack of thyroid hormone is associated with cone photoreceptor preservation, while administration of high doses of active thyroid hormone leads to deterioration. The association between thyroid function and age-related macular degeneration (AMD) has not been investigated in the general population.

**Methods:**

Participants of age ≥55 years from the Rotterdam Study with thyroid-stimulating hormone (TSH) and/or free thyroxine (FT4) measurements and AMD assessment were included. We conducted age- and sex-adjusted Cox proportional hazards models to explore the association of TSH or FT4 with AMD, in the full range and in those with TSH (0.4-4.0 mIU/L) and/or FT4 in normal range (11–25 pmol/L). Cox proportional hazards models were performed for the association of TSH or FT4 with retinal pigment alterations (RPA), as an early marker of retinal changes. Multivariable models additionally included cardiovascular risk factors and thyroid peroxidase antibodies positivity. We also performed stratification by age and sex. A bidirectional look-up in genome-wide association study (GWAS) data for thyroid parameters and AMD was performed. Single nucleotide polymorphisms (SNPs) that are significantly associated with both phenotypes were identified.

**Results:**

We included 5,573 participants with a median follow-up of 6.9 years (interquartile range 4.4-10.8 years). During follow-up 805 people developed AMD. TSH levels were not associated with increased risk of AMD. Within normal range of FT4, participants in the highest FT4 quintile had a 1.34-fold increased risk of developing AMD, compared to individuals in the middle group (95% confidence interval [CI] 1.07-1.66). Higher FT4 values in the full range were associated with a higher risk of AMD (hazard ratio 1.04, CI, 1.01-1.06 per 1 pmol/L increase). Higher FT4 levels were similarly associated with a higher risk of RPA. Restricting analyses to euthyroid individuals, additional multivariable models, and stratification did not change estimates. We found a SNP (rs943080) in the *VEGF-A* gene, associated with AMD, to be significant in the TSH GWAS (*P* = 1.2 x 10^−4^). Adding this SNP to multivariable models did not change estimates.

**Conclusions:**

Higher FT4 values are associated with increased risk of AMD - even in euthyroid individuals - and increased risk of RPA. Our data suggest an important role of thyroid hormone in pathways leading to AMD.

**Electronic supplementary material:**

The online version of this article (doi:10.1186/s12916-015-0329-0) contains supplementary material, which is available to authorized users.

## Background

Age-related macular degeneration (AMD) is a disease of the retina in the elderly which can lead to irreversible blindness and is characterized by drusen, pigmentary changes, choroidal neovascularization, and geographic atrophy. While AMD is one of the leading causes of visual impairment worldwide and increasing in prevalence [1-7], the exact pathophysiology and pathways leading to AMD are not entirely understood. Thyroid hormones are known to regulate various visual functions in experimental and human studies [8-10]. Human retinal pigment epithelial (RPE) cells express thyroid hormone receptors and seem to be a direct target for thyroid hormones [11]. Recently it was shown that suppression of thyroid hormone signaling resulted in preservation of cone photoreceptors in mouse models of retinal degeneration [12]. In contrast, administration of active thyroid hormone leads to deterioration of cones. Thyroid dysfunction and subclinical thyroid dysfunction are common in the general population, with a prevalence up to 10% [13-16]. These thyroid disorders are associated with various cardiovascular risk factors, including alterations in lipid levels, atherosclerosis, and hypertension [17-19], which are known predisposing factors for development and progression of AMD [20,21]. However, there are no studies in the general population assessing the association between thyroid function and the risk of AMD. Therefore, we aimed to assess the relation between thyroid-stimulating hormone (TSH), free thyroxine (FT4), and the risk of incident AMD in a prospective population-based cohort study and to study possible underlying genetic pathways by investigating an overlap in genome-wide significant hits (that is, bidirectional genetic look-up).

## Methods

### The Rotterdam Study

The Rotterdam Study is a prospective population-based cohort study that addresses determinants and occurrence of cardiovascular, neurological, ophthalmologic, psychiatric, and endocrine diseases in the elderly living in Ommoord, a suburb of Rotterdam. The aims and design of the Rotterdam study have been described in detail elsewhere [22]. For this analysis we included participants from two independent cohorts from the Rotterdam Study. The Rotterdam Study Cohort 1 (RSI) started in 1989 and included a total of 7,983 participants (response rate 78%) aged 55 years and older. Baseline data were collected from 1990 until 1993, and four follow-up examinations were performed in 1993–1995, 1997–1999, 2002–2004, and 2009–2011. The second cohort is the Rotterdam Study Cohort II (RSII), which includes a total of 3,011 participants (response rate 67%) aged 55 years and older. Baseline data were collected from 2000–2001, and follow-up examinations were performed in 2004–2005 and 2011–2012.

### Study population

Participants from baseline study cohorts RSI (RSI-1) and RSII (RSII-1) were eligible for these analyses if they had TSH and/or FT4 measurements and had gradable fundus photographs at baseline and at least one follow-up eye examination. Since not all participants from RSI had thyroid measurements at baseline, additional baseline samples were drawn from RSI visit 3 (RSI-3). Participants with AMD at baseline (N = 567) were excluded from further analyses. In total, 5,573 participants from these two cohorts were eligible to be included in our analyses (Additional file 1: Figure S1). The Medical Ethics Committee of the Erasmus University approved the study protocols, and participants gave a written informed consent in accordance with the Declaration of Helsinki.

### Assessment of thyroid function

For RSI-1, serum TSH (TSH Lumitest; Henning, Berlin, Germany), anti-TPOAb (ELISA; Milenia; Diagnostic Products Corp, Los Angeles, CA, USA) and free T4 levels (FT4; Vitros, ECI Immunodiagnostic System; Ortho-Clinical Diagnostics, Amersham, UK) were determined in a random subset of the baseline serum samples (n = 1,855). Thyroid function assessment was also performed in baseline serum samples for TSH and FT4 (electrochemiluminescence immunoassay for thyroxine and thyrotropine, “ECLIA”, Roche) for RSI-3 and RSII-1. The tests’ TSH reference ranges did not differ substantially and had a good Spearman correlation coefficient (0.96 for TSH, *P* < 0.0001 and 0.81 for FT4, *P* < 0.0001). We determined the cut-off values for normal range TSH as 0.4-4.0 mIU/L according to national guidelines. The reference range for FT4 was 11–25 pmol/L, and anti-TPOAb levels greater than 60 kU/mL were regarded as positive.

### Diagnosis of age-related macular degeneration

All eligible participants underwent fundus photography after pharmacologic mydriasis. For visits RSI-1 to RSI-3 and RSII-1 a 35° film fundus camera was used (Topcon TRV-50VT, Topcon Optical Company, Tokyo, Japan), after which a 35° digital color fundus camera (Topcon TRC-50EX, Topcon Optical Company, Tokyo, Japan with a Sony DXC-950P digital camera; 0.44 megapixel, Sony Corporation, Tokyo, Japan) followed for visits RSI-4, RSI-5, RSII-2, and RSII-3. Fundus transparencies were graded according to the Wisconsin age-related maculopathy grading system [23] and the modified International Classification System [24] by trained graders under the supervision of senior retinal specialists (JRV, CCWK). The eyes of each participant were graded and classified separately, and the eye with the more severe grade was used to classify the person. In the analyses incident early and late AMD combined was used as the outcome variable. In the manuscript this is referred to as AMD. Besides AMD we also investigated AMD-specific lesions as a separate outcome variable. These lesions included retinal pigmentary alterations, large drusen (≥125 μm), and large drusen area (≥5,331,820 μm^2^) [25].

### Baseline measurements

Smoking was derived from computerized baseline questionnaires, and participants were categorized as current or non-current smokers. Blood pressure, systolic and diastolic, was calculated as the average of two consecutive measurements, using random-zero mercury sphygmomanometers. Hypertension was defined as a systolic blood pressure ≥ 140 mmHg or a diastolic blood pressure ≥ 90 mmHg or participant use of anti-hypertensive medication at baseline. Cholesterol was measured at baseline by the CKCL (Centra Clinical Chemical Laboratory) of the Erasmus University Medical Center. A subgroup of measurements was carried out in the laboratory of the Department of Epidemiology and Biostatistics (Erasmus University Medical School). History of diabetes was defined by a repeated impaired fasting glucose ≥ 7 or use of anti-glycemic medication at baseline. Body mass index (BMI) was calculated as weight in kilograms divided by height in meters squared.

### Statistical analysis

Participant baseline characteristics were compared using a χ^2^ or *t*-test. Due to a skewed distribution, TSH was log-transformed for the statistical analyses. We used the Cox proportional hazards model to calculate the relationship between TSH and FT4 at baseline and the risk of incident AMD, first including all participants and then including only those with normal range TSH and/or FT4 values. We performed a crude Cox model including only thyroid parameters, after which we also included quadratic and cubic terms to explore possible nonlinear relationships. We then performed additional models adjusting first for age and sex and second also adding smoking, hypertension, cholesterol, diabetes, and BMI to the model. Hypertension, cholesterol, diabetes, and BMI could act as confounders and possible mediators depending on the presumed pathway through which thyroid function is related to AMD. These variables were included in the multivariable model as possible confounders of non-vascular pathways. We looked at the association between AMD and TSH or FT4 both continuously and in quintiles, as well as overall and within the normal range of TSH. The middle quintile was used as a reference group, as biologically it is expected to represent the subgroup with the most normal thyroid function within the euthyroid group. We performed predefined stratification by sex and age categories, using a cutoff of 65 years, as this is the median of the current population and the treatment threshold for subclinical thyroid dysfunction according to the European guidelines [26]. Further interaction terms were introduced to the model to explore possible differential risk patterns. We performed a sensitivity analysis excluding those using thyroid medication at baseline (levothyroxine and anti-thyroid drugs) and those with prior self-reported thyroid disease at baseline. We also performed FT4 and TSH analyses with specific AMD lesions defined as retinal pigment alteration, large drusen, and large drusen area as separate outcome variables to examine possible early changes in underlying pathways. To address the issue of drop-out of individuals during follow-up that could possibly not be completely at random, we adjusted the model for inverse probability weights (IPWs). These were calculated using possible baseline explanatory variables for drop-out such as smoking, BMI, and medication use. The proportional hazards assumption was checked statistically using the Schoenfeld test and assessing the Schoenfeld plot. All statistical analyses were performed using SPSS version 21 (IBM SPSS, Armonk, NY, USA) except for the Schoenfeld tests and (Schoenfeld) plots which were performed in R (survival package, R-project, Institute for Statistics and Mathematics, R Core Team (2013), Vienna, Austria, version 3.0.2).

### Bidirectional genetic look-up

Genome-wide association studies (GWASs) have been performed for AMD [27] and thyroid function (TSH and FT4) [28,29]. These studies identified several single nucleotide polymorphisms (SNPs) associated to these two phenotypes. Some of the genome-wide significant SNPs in the AMD GWAS might also play a role in thyroid function and vice versa. Overlap between common genetic polymorphisms can provide insight into possible shared genetic pathways. It might also elucidate a mediation effect between the two phenotypes, that is, identify and explicate the process that underlies a possible observed relationship between thyroid function and AMD. To evaluate these potential genetic pathways, we conducted a bidirectional genetic look-up using the results of the above mentioned GWASs for AMD and thyroid function. We first extracted SNPs that reached genome-wide significance from the AMD GWAS performed by the AMD Gene Consortium [27]. We then checked whether these were significantly associated with TSH or FT4 in the thyroid function GWAS performed by Porcu *et al*. [28]. Hereafter we extracted the genome-wide significant SNPs for TSH or FT4 from the thyroid function GWAS and checked whether they were associated with AMD in the AMD GWAS. For the significance level, we applied a multiple testing correction (Bonferroni correction), using a *P*-value threshold of 0.05 divided by the amount of significant SNPs per GWAS. In case of a significant finding, we added the SNP to the multivariable model to evaluate a possible mediation effect.

## Results

We included 5,573 participants with TSH and/or FT4 measurements at baseline and incident AMD data, with a median follow-up of 6.9 years (interquartile range [IQR] of 4.4-10.8 years). Of these, 5,572 had TSH and 5,504 had FT4 baseline measurements. A total of 805 people developed AMD (early AMD N = 725, late AMD N = 80) during follow-up with an incidence rate of 18 per 1,000 person-years. The baseline characteristics for those with and without incident AMD during follow-up were comparable, except for the proportion of diabetes (Table 1).

**Table 1:** Baseline characteristics of included participants from the Rotterdam Study evaluating the association between thyroid function and AMD*

**Variable**	**No incident AMD**	**Incident AMD**	
	**N = 4,768**	**N = 805**	***P*** -value**
Age, years	67.6 (7.6)	67.9 (7.1)	0.29
Sex, % female	57.6	57.8	0.94
History of diabetes, %	10.8	8.4	0.04
BMI, kg/m^2^	26.9 (3.9)	26.6 (3.7)	0.07
Cholesterol, mmol/L	6.1 (1.2)	6.1 (1.1)	0.23
Smoking, % current	20.7	21.0	0.85
Hypertension, %	63.0	58.7	0.17
TSH, mIU/L median (IQR)	1.78 (1.15-2.69)	1.73 (1.17-2.67)	0.78
FT4, pmol/L	15.8 (2.6)	16.0 (3.2)	0.13
TPOAb, kU/L	30.5 (95.1)	30.8 (96.2)	0.93

*Values are means and SD unless otherwise specified.

**For comparison a *t*-test was conducted, for TSH the log-transformed values were used.

*Abbreviations: AMD* age-related macular degeneration *BMI* body mass index, *TSH* thyroid-stimulating hormone, FT4 = free thyroxine, *SD* standard deviation, *IQR* interquartile range, *TPOAb* thyroid peroxidase antibodies.

### Association between thyroid function and AMD

**Figure 1:**
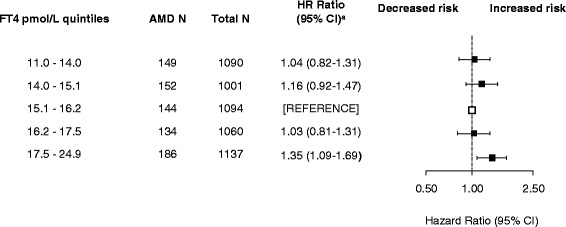
Quintiles of FT4 within the normal range and risk of AMD. The normal range of FT4 was defined as 11–25 pmol/L (conversion 1 pmol/L = 0.0777 ng/dL). ^a^Analyses were adjusted for sex, age, smoking, hypertension, cholesterol, diabetes, body mass index, and thyroid peroxidase antibodies positivity. Abbreviations: AMD, age-related macular degeneration; FT4, free thyroxine; HR, hazard ratio.

Although there was no association between TSH and AMD (hazard ratio [HR] 0.99; 95% confidence interval [CI] 0.91-1.07, Table 2), the risk of AMD was significantly increased in those with higher FT4 levels (Table 2). When categorizing the FT4 values within normal range in quintiles, those in the highest FT4 quintile had an increased risk compared to the middle group with an HR of 1.34 (95% CI, 1.07-1.66) and a non-significant *P* for interaction (*P* = 0.066) (Table 2). This association remained similar after additional adjustments for smoking, diabetes, hypertension, cholesterol, BMI, and TPOAb positivity (Figure 1). This association also remained similar after analyzing only those within the normal range of TSH and FT4, that is, those with normal thyroid function. Excluding those with thyroid medication or thyroid disease at baseline as a sensitivity analysis did not alter the association (Table 3). Stratifying for age and sex did not reveal any significant differential risk (Additional file 1: Table S1). The association between thyroid function and retinal pigment alterations for FT4 showed similar significant HRs, with the exception of the risk estimates when looking at FT4 only in the normal range of TSH (Table 4). TSH and FT4 were not associated with large drusen or large drusen area (data not shown). Introducing quadratic and cubic terms for TSH and FT4 to the crude model, as an exploration of non-linearity, did not improve model performance. Taking possible non-random follow-up using IPWs did not change risk estimates. The proportional hazards assumption was checked statistically with the Schoenfeld test and Schoenfeld plot and met for both the TSH (*P* = 0.232) and FT4 (*P* = 0.154) analyses.

**Table 2:** **Association between TSH, FT4, and risk of AMD**

**Incident AMD versus no AMD**	**AMD** ***N***	**Total** ***N***	**HR (95% CI), model 1**	**HR (95% CI), model 2**	**HR (95% CI), model 3**
*TSH, mIU/L*	805	5,572	0.99 (0.91-1.07)	0.99 (0.91-1.07)	0.99 (0.91-1.07)
*TSH in normal range* ^a^	696	4,756	1.06 (0.91-1.23)	1.09 (0.93-1.27)	1.08 (0.93-1.26)
*Normal range TSH* ^a^					
Q1 0.40-1.10	148	1,082	1.04 (0.82-1.32)	1.00 (0.79-1.28)	1.00 (0.79-1.28)
Q2 1.11-1.54	167	990	1.32 (1.04-1.66)	1.29 (1.02-1.62)	1.29 (1.02-1.62)
Q3 1.55-1.99	128	962	reference	reference	reference
Q4 2.00-2.61	117	851	1.09 (0.85-1.40)	1.07 (0.83-1.37)	1.07 (0.83-1.38)
Q5 2.62-3.97	136	871	1.22 (0.96-1.56)	1.22 (0.95-1.55)	1.21 (0.94-1.54)
*P interaction*			*0.648*	*0.485*	*0.517*
*Total*	*696*	*4,756*			
*FT4, pmol/L*	791	5,504	1.04 (1.01-1.06)	1.04 (1.01-1.06)	1.04 (1.01-1.06)
*FT4 in normal range* ^b^	765	5,382	1.04 (1.01-1.07)	1.04 (1.01-1.07)	1.04 (1.01-1.07)
*Normal range FT4* ^b^					
Q1 11.0-14.0	149	1,090	1.03 (0.82-1.29)	1.04 (0.82-1.31)	1.04 (0.82-1.31)
Q2 14.0-15.1	152	1,001	1.12 (0.89-1.41)	1.17 (0.92-1.47)	1.16 (0.92-1.47)
Q3 15.1-16.2	144	1,094	reference	reference	reference
Q4 16.2-17.5	134	1,060	1.01 (0.80-1.28)	1.03 (0.81-1.30)	1.03 (0.81-1.31)
Q5 17.5-24.9	186	1,137	1.34 (1.07-1.66)	1.35 (1.08-1.69)	1.35 (1.09-1.69)
*P interaction*	*765*	*5,382*	*0.066*	*0.088*	*0.080*
*Normal range FT4* ^b^ *in normal range TSH* ^a^	673	*4,658*	1.04 (1.01-1.08)	1.04 (1.01-1.08)	1.04 (1.01-1.07)

^a^Normal range of TSH defined as 0.4-4.0 mIU/L. ^b^Normal range of FT4 defined as 11–25 pmol/L.

Model 1: Adjusted for sex and age. Model 2: Model 1 + smoking, hypertension, cholesterol, diabetes, BMI. Model 3: Model 2 + thyroid peroxidase antibodies positivity.

*Abbreviations: AMD* age-related macular degeneration, *BMI* body mass index, *CI* confidence interval, *FT4* free T4, *HR* hazard ratio, *Q* quintile, *TSH* thyroid-stimulating hormone.

**Table 3:** **Sensitivity analyses excluding participants with thyroid medication or thyroid disease at baseline**

**Incident AMD versus no AMD**	**AMD N**	**Total N**	**HR (95% CI), model 1**	**HR (95% CI), model 2**	**HR (95% CI), model 3**
Excluded thyroid medication					
Free T4	752	5,225	1.03 (1.01-1.06)	1.03 (1.01-1.06)	1.03 (1.01-1.06)
TSH, mIU/L	778	5,417	0.99 (0.91-1.08)	1.00 (0.92-1.09)	1.00 (0.91-1.09)
Excluding baseline thyroid disease^b^					
Free T4	751	5,237	1.04 (1.01-1.08)	1.04 (1.01-1.07)	1.04 (1.01-1.08)
TSH, mIU/L	764	5,300	0.98 (0.89-1.07)	0.98 (0.89-1.07)	0.97 (0.89-1.07)

^a^155 participants had thyroid medication (thyroid hormone use) at baseline.

^b^272 participants had self-reported thyroid disease at baseline.

Model 1: Adjusted for sex and age. Model 2: Model 1 + smoking, hypertension, cholesterol, diabetes, BMI. Model 3: Model 2 + thyroid peroxidase antibodies positivity.

*Abbreviations: BMI* body mass index, *CI* confidence interval, *FT4* free thyroxine, *HR* hazard ratio, *TSH* thyroid-stimulating hormone.

**Table 4:** **Association between FT4 and TSH with retinal pigment alterations** ^**a**^

**Incident pigment alterations versus no pigment alterations**	**Cases N**	**Total N**	**HR (95% CI), model 1**	**HR (95% CI), model 2**	**HR (95% CI), model 3**
TSH, mIU/L	729	5,401	0.98 (0.90-1.06)	0.97 (0.90-1.06)	0.96 (0.88-1.04)
Normal range TSH^b^	618	4,591	1.02 (0.87-1.20)	1.05 (0.89-1.23)	1.04 (0.88-1.22)
FT4, pmol/L	720	5,338	1.04 (1.01-1.07)	1.04 (1.01-1.07)	1.04 (1.01-1.07)
Normal range FT4^c^	697	5,226	1.04 (1.01-1.07)	1.04 (1.01-1.07)	1.04 (1.01-1.07)
Normal range FT4^c^ in normal range TSH^b^	601	4,500	1.03 (1.00-1.07)	1.03 (0.99-1.06)	1.03 (0.99-1.06)

^a^Participants with late AMD were excluded from this analysis.

^b^Normal range of TSH defined as 0.4-4.0 mIU/L.

^c^Normal range of FT4 defined as 11–25 pmol/L.

Model 1: Adjusted for sex and age. Model 2: Model 1 + smoking, hypertension, cholesterol, diabetes, BMI. Model 3: Model 2 + thyroid peroxidase antibodies positivity.

*Abbreviations: BMI* body mass index, *CI* confidence interval, *FT4* free thyroxine, *HR* hazard ratio, *TSH* thyroid-stimulating hormone.

### Bidirectional genetic look-up

In the thyroid function GWAS, 20 SNPs were associated with TSH and 6 with FT4 [28]. The AMD GWAS revealed 19 genome-wide significant SNPs related to the phenotype. None of the SNPs from the thyroid function GWAS were significant in the AMD GWAS. One SNP (rs943080) in the vascular endothelial growth factor A (*VEGF-A*) gene that is related to AMD was also significantly associated with TSH (*P* = 1.2 × 10^−4^, significance threshold =0.0026) (Additional file 1: Table S2). Within our study population, GWAS data were available for a total 4,646 participants. Additionally correcting for the rs943080 SNP in the most adjusted model in these participants resulted in similar risk estimates for the FT4 analysis (HR 1.04, CI 95% 1.01-1.07). Stratifying for this SNP did show risk differences between the different genotypes but not significantly (Additional file 1: Table S1).

## Discussion

In this prospective cohort study we investigated the association between thyroid function and incidence of AMD. Higher FT4 values were associated with an increased risk of developing AMD, even within the normal range of TSH and FT4 (that is, euthyroid subjects), while there was no association between TSH and AMD. The similar findings between higher FT4 levels and retinal pigment alterations might suggest that thyroid hormone plays a role in the development of AMD rather than just acting as a promoter of disease. To our knowledge, this is the first prospective population-based cohort study to look at the association between thyroid function and AMD.

A limited number of studies investigating thyroid disease and AMD have been published, all lacking laboratory assessment of thyroid function. Bromfield *et al*. reported an increased risk of AMD in subjects with self-reported hypothyroidism [30]. A case–control study by Anand *et al*. reported an association between thyroid hormone use and a higher risk of AMD with geographic atrophy [31], but no data were reported on the number of patients that were over- or undertreated. Similarly, the Beaver Dam Eye study also reported an association between thyroid hormone use and early AMD [32], but this was not confirmed by Douglas *et al*. [33]. As mentioned previously, none of these studies had laboratory assessment of thyroid function nor did they investigate the association in a time-to-event analysis. In our study, excluding all subjects using thyroid medication did not alter risk estimates, supporting a potential intrinsic effect of thyroid hormone.

There are several pathophysiological explanations for the relationship between thyroid hormones and AMD. In a mouse model of retinal degeneration, suppression of thyroid hormone signaling resulted in preservation of cone photoreceptors [12]. The same study found that stimulating thyroid hormone signaling, by administering the active thyroid hormone triiodothyronine, deteriorates cones in mouse models with a slow progressive and moderate degeneration phenotype [12]. In addition, mice lacking type 3 deiodinase, the enzyme responsible for the degradation of thyroid hormones, have decreased survival and disturbed maturation of cone photoreceptors [34]. The findings of these studies suggest that thyroid hormone may lead to a higher turnover of photoreceptors, and in retinal degeneration this leads to deterioration of photoreceptors. Beside photoreceptors, thyroid hormone might also have an influence on the retinal pigment epithelial cells [11]. In the healthy retina the turnover of photoreceptors is extremely high. Every day the photoreceptors shed the ends of their outer segments, resulting in full renewal every 10 days. These shedded parts of the outer segments are phagocytosed by the retinal pigment epithelium (RPE) cells [35]. Increase of the turnover of the photoreceptors by thyroid hormone may bring additional stress to the process. RPE cells at distress may change, resulting in pigmentary alterations in the macular area. The RPE cells may also be targeted directly by the thyroid hormone, resulting in these changes [11]. These results may provide an explanation for the findings in our study.

Thyroid dysfunction has been linked to cardiovascular risk factors and disease, including effects on the vascular function, lipids, and atherosclerosis [36]. As some of these risk factors are also linked to AMD [20,21], one could speculate about a joint vascular pathway leading to both thyroid dysfunction and AMD or perhaps that the relation between thyroid dysfunction and AMD could be mediated through this pathway. We were not able to confirm these hypotheses. First of all, these cardiovascular risk factors are mainly seen in hypothyroidism, (high TSH and low FT4), whereas our data show an association between high FT4 and AMD. Also, correcting for some of these risk factors (for example, hypertension) that could act as confounders and possible mediators did not change risk estimates, suggesting that the effect of thyroid function is not through these pathways. Lastly, the *VEGF-A* gene was found to be significant in the look-up for the TSH GWAS and not the FT4 GWAS. However, our results suggest a higher risk of AMD in higher levels of FT4 and not in TSH. Furthermore, the association did not change by adding this SNP to the multivariable model.

We find an effect with FT4 but not with TSH, which seems to be in line with previous literature from cohort studies in elderly populations investigating the relation between thyroid function and several other endpoints [37,38]. Regulation of serum thyroid hormone levels is controlled by the hypothalamus-pituitary-thyroid axis. The set point of this feedback mechanism is defined individually, with thyroid hormone levels showing a much greater inter-individual than intra-individual variability [39]. The individual set point can be modulated by several pathophysiological (for example, critical illness) and physiological (for example, ageing) mechanisms [40]. This could explain why in this elderly and ageing population we do find an association with FT4 but not with TSH, especially in the euthyroid range. Furthermore, previous literature results showed an increase in TSH with increasing age, suggesting that higher TSH levels are needed to keep thyroid hormone levels within the desired range [38]. We only have thyroid function measures at baseline and are therefore not able to investigate whether changes in thyroid function over time explain the discordant association between TSH, FT4, and AMD.

Important strengths of our study are the assessment of thyroid function at baseline through laboratory testing as well as the elaborate assessment of AMD at baseline and follow-up. Also, we were able to investigate the association between thyroid function and specific AMD lesions like retinal pigment alterations to examine possible early changes in underlying pathways. The availability of genetic data gave us the opportunity to explore possible genetic pathways. The bidirectional genetic look-up, revealed one SNP in the *VEGF-A* gene to be significant in the TSH GWAS, but not for FT4. Adding this SNP to the multivariate model did not alter risk estimates. An explanation for the absence of overlapping genome-wide significant SNPs could be that these GWASs were underpowered for this association.

A limitation of our study is that thyroid parameters were measured once at baseline. Therefore, the evolution of thyroid hormone levels could not be taken into account. Also, residual confounding cannot be excluded, even with the large number of covariates included in these analyses. Lastly, this study is conducted in a mainly Caucasian population of 55 years and older and may not be generalizable to other populations.

## Conclusions

We find an increased risk of incident AMD in subjects with higher FT4 levels, even in those with a normal thyroid function and when excluding thyroid medication users. This implies an intrinsic (that is, not exogenous) deleterious effect of thyroid hormone on AMD. We also find an association between higher FT4 levels and retinal pigment alterations, suggesting that thyroid hormone could even play a role in the early stage of development of AMD. Functional and clinical studies could provide more evidence for a true causal relationship.

## Additional file

Additional file 1: Figure S1. Flow chart for inclusion of participants from the Rotterdam Study. Abbreviations: AMD, age-related macular degeneration; RS, Rotterdam Study. **Table S1a.** Stratification analysis of the association between TSH in normal range and risk of AMD. **Table S1b.** Stratification analysis of the association between FT4 in normal range TSH and risk of AMD. **Table S2a.** Associations of genome-wide significant AMD hits with TSH and FT4 levels; look-up in TSH-FT4 GWAS. **Table S2b.** Association of genome-wide significant TSH and FT4 SNPS with late AMD; look-up in AMD GWAS.
