# Supplementary material for: Thyroid function and age-related macular degeneration: a prospective population-based cohort study - the Rotterdam Study
Source: BMC Med. 2015 Apr 23;13:94. doi: 10.1186/s12916-015-0329-0 (PMC4407352; doi:10.1186/s12916-015-0329-0)
Supplement: Additional file 1: Figure S1. — Flow chart for inclusion of participants from the Rotterdam Study. Abbreviations: AMD, age-related macular degeneration; RS, Rotterdam Study. Table S1a. Stratification analysis of the association between TSH in normal range and risk of AMD. Table S1b. Stratification analysis of the association between FT4 in normal range TSH and risk of AMD. Table S2a. Associations of genome-wide significant AMD hits with TSH and FT4 levels; look-up in TSH-FT4 GWAS. Table S2b. Association of genome-wide significant TSH and FT4 SNPS with late AMD; look-up in AMD GWAS. [file 12916_2015_329_MOESM1_ESM.docx]

**ADDITIONAL DATA**

**Additional Figure legend**

Additional Figure 1
Flow chart for inclusion of participants from the Rotterdam Study
Abbreviations: AMD Age-related Macular Degeneration ; RS Rotterdam Study

**Additional Figure 1**

Participants in RSI & RSII
(n =10,994)

Excluded participants with missing thyroid function measurements
(n=4064)

Thyroid function available (random selection of participants) in RSI & RSII
(n =6930)

No baseline fundus photo’s (n=294) or prevalent AMD (n = 567)

Remaining number of participants RSI & RSII
(n =6069)

Excluded due to missing follow-up data
(n = 496)

Final number of participants included from RSI & RSII
(n =5573)

| **Additional Table 1a Stratification analysis of the association between TSH in normal range and risk of AMD** | | | | | |
| --- | --- | --- | --- | --- | --- |
| **Incident AMD vs no AMD** | **AMD N** | **Total N** | **HR (95% CI), model 1** | **HR (95% CI), model 2** | **HR (95% CI), model 3** |
|  |  |  |  |  |  |
| **Sex^a^** |  |  |  |  |  |
| Males | 316 | 2144 | 1.04 (0.90-1.20) | 1.04 (0.90-1.24) | 1.06 (0.91-1.24) |
| Females | 380 | 2612 | 0.97 (0.88-1.06) | 0.97 (0.88-1.03) | 0.96 ( 0.87-1.05) |
| *Total* | *696* | *4756* |  |  |  |
| *P interaction = 0.385* |  |  |  |  |  |
|  |  |  |  |  |  |
| **Age categories^b^** |  |  |  |  |  |
| 45 – 65 years | 270 | 2043 | 1.02 (0.89-1.17) | 1.03 (0.93-1.19) | 1.02 (0.89-1.17) |
| >65 years | 426 | 2713 | 0.97 (0.88-1.07) | 0.96 (0.83-1.07) | 0.97 (0.87-1.07) |
| *Total* | *696* | *4756* |  |  |  |
| *P interaction = 0.368* |  |  |  |  |  |
|  |  |  |  |  |  |
| ***VEGFA* rs943080** |  |  |  |  |  |
| C/C | 132 | 1002 | 0.98 (0.82-1.18) | 0.98 (0.82-1.18) | 0.99 (0.82-1.19) |
| T/C | 296 | 2038 | 1.00 (0.89-1.13) | 1.01 (0.89-1.15) | 1.02 (0.89-1.16) |
| T/T | 175 | 1003 | 0.94 (0.81-1.09) | 0.95 (0.81-1.10) | 0.94 (0.81-1.10) |
| *Total* | *603* | *4043* |  |  |  |
| *P interaction = 0.669* |  |  |  |  |  |
| Model 1: Adjusted for sex and age. Model 2: Model 1 + smoking, hypertension, cholesterol, diabetes, BMI. Model 3: Model 2 + thyroid peroxidase antibodies positivity  ^a^These analyses were not additionally adjusted for sex. ^b^ These analyses were adjusted for sex and age as continuous variable to avoid residual confounding within age strata.  Abbreviations: AMD Age-related Macular Degeneration;TSH thyroid-stimulating hormone; FT4 free thyroxine; BMI body-mass index; CI confidence interval; HR hazard ratio | | | | | |

| **Additional Table 1b: Stratification analysis of the association between FT4 in normal range TSH and risk of AMD** | | | | | |
| --- | --- | --- | --- | --- | --- |
| **Incident AMD vs no AMD** | **AMD N** | **Total N** | **HR (95% CI), model 1** | **HR (95% CI), model 2** | **HR (95% CI), model 3** |
|  |  |  |  |  |  |
| **Sex^a^** |  |  |  |  |  |
| Males | 310 | 2116 | 1.04 (0.99-1.09) | 1.04 (0.99-1.09) | 1.04 (0.99-1.08) |
| Females | 374 | 2588 | **1.04 (1.01-1.07)** | **1.04 (1.01-1.07)** | **1.04 (1.01-1.07)** |
| *Total* | *684* | *4704* |  |  |  |
| *P interaction = 0.875* |  |  |  |  |  |
|  |  |  |  |  |  |
| **Age categories^b^** |  |  |  |  |  |
| 45 – 65 years | 266 | 2027 | **1.05 (1.02-1.09)** | **1.05 (1.01-1.09)** | **1.05 (1.02-1.09)** |
| >65 years | 418 | 2677 | 1.03 (0.99-1.06) | 1.02 (0.99-1.06) | 1.02 (0.99-1.06) |
| *Total* | *684* | *4704* |  |  |  |
| *P interaction = 0.164* |  |  |  |  |  |
|  |  |  |  |  |  |
| ***VEGFA* rs943080** |  |  |  |  |  |
| C/C | 132 | 994 | **1.06 (1.01-1.13)** | 1.05 (0.99-1.12) | 1.05 (0.99-1.12) |
| T/C | 290 | 2011 | 1.02 (0.98-1.06) | 1.01 (0.97-1.05) | 1.01 (0.91-1.05) |
| T/T | 172 | 995 | **1.07 (1.03-1.11)** | **1.07 (1.03-1.11)** | **1.070(1.03-1.11)** |
| *Total* | *594* | *4000* |  |  |  |
| *P interaction = 0.561* |  |  |  |  |  |
| Model 1: Adjusted for sex and age. Model 2: Model 1 + smoking, hypertension, cholesterol, diabetes, BMI. Model 3: Model 2 + thyroid peroxidase antibodies positivity  ^a^These analyses were not additionally adjusted for sex.  ^b^ These analyses were adjusted for sex and age as continuous variable to avoid residual confounding within age strata.  Abbreviations: AMD Age-related Macular Degeneration; TSH thyroid-stimulating hormone; FT4 free thyroxine; BMI body-mass index; CI confidence interval; HR hazard ratio | | | | | |

| **Additional Table 2a. Associations of genome-wide significant AMD hits^a^ with TSH and FT4 levels; look-up in TSH-FT4 GWAS^b^** | | | | | | | | | |
| --- | --- | --- | --- | --- | --- | --- | --- | --- | --- |
|  |  |  |  | ***TSH*** | | | ***FT4*** | | |
| **Gene** | **SNP** | **Allele1** | **Allele2** | **Direction of β** | **P value** | **N** | **Direction of β** | **P value** | **N** |
| *ARMS2-HTRA1* | rs10490924 | T | G | + | 0.07022 | 25928 | - | 0.3038 | 17391 |
| *CFH* | rs10737680 | A | C | + | 0.9935 | 25947 | + | 0.653 | 17410 |
| *C2-CFB* | rs429608 | A | G | - | 0.4336 | 23810 | - | 0.6777 | 15272 |
| *C3* | rs2230199 | C | G | + | 0.3497 | 14088 | - | 0.2693 | 8709 |
| *TIMP3* | rs5749482 | C | G | - | 0.5259 | 24569 | - | 0.7798 | 16031 |
| *APOE* | rs4420638 | A | G | + | 0.8079 | 17791 | + | 0.6501 | 12412 |
| *CETP* | rs1864163 | A | G | - | 0.7363 | 18055 | + | 0.8782 | 9517 |
| ***VEGFA*** | **rs943080** | **T** | **C** | **-** | **1.2x10^-4^** | **20405** | **-** | **0.3426** | **11868** |
| *TNFRSF10A* | rs13278062 | T | G | - | 0.2557 | 19234 | + | 0.3095 | 10696 |
| *LIPC* | rs920915 | C | G | + | 0.7489 | 25481 | + | 0.7276 | 16943 |
| *CFI* | rs4698775 | T | G | - | 0.5895 | 25763 | - | 0.2744 | 17225 |
| *COL10A1* | rs3812111 | A | T | - | 0.5249 | 23559 | + | 0.7403 | 17155 |
| *COL8A1-FILIP1L* | rs13081855 | T | G | - | 0.08712 | 26053 | - | 0.613 | 17516 |
| *IER3-DDR1* | rs3130783 | A | G | - | 0.735 | 22832 | + | 0.1474 | 14294 |
| *SLC16A8* | rs8135665 | T | C | + | 0.2493 | 20771 | + | 0.1255 | 13259 |
| *TGFBR1* | rs334353 | T | G | + | 0.06685 | 25054 | + | 0.08443 | 16517 |
| *RAD51B* | rs8017304 | A | G | + | 0.4073 | 26055 | + | 0.4401 | 17518 |
| *ADAMTS9* | rs6795735 | T | C | - | 0.3906 | 25760 | - | 0.5288 | 17222 |
| *B3GALTL* | rs9542236 | T | C | - | 0.8603 | 25978 | - | 0.2864 | 17441 |
| Abbreviations: AMD age-related macular degeneration; TSH thyroid-stimulating hormone; FT4 free thyroxine, β = β coefficient found in the Thyroid function GWAS; SNP Single Nucleotide Polymorphisms  ^a^ Fritsche LG et al, Nature genetics, 2013  ^b^ Porcu E et al. PLOS genetics, 2013 | | | | | | | | | |

| **Additional Table 2b. Association of genome-wide significant TSH and FT4^a^ SNPS with late AMD; look-up in AMD GWAS^b^** | | | | | | | |
| --- | --- | --- | --- | --- | --- | --- | --- |
| **Gene** | **SNP** | **Allele1** | **Allele2** | **Direction of β** | **P value** | **Ncases** | **Ncontrols** |
| **TSH** |  |  |  |  |  |  |  |
| *PDE8B* | rs6885099 | A | G | - | 0.75391 | 7647 | 51729 |
| *PDE10A* | rs753760 | C | G | + | 0.014564 | 7538 | 51607 |
| *CAPZB* | rs10799824 | A | G | + | 0.98795 | 7648 | 51728 |
| *MAF/LOC440389* | rs3813582 | T | C | - | 0.68101 | 7647 | 51718 |
| *VEGFA* | rs9472138 | T | C | + | 0.003572 | 7648 | 51725 |
| *VEGFA* | rs11755845 | T | C | - | 0.16559 | 7647 | 51700 |
| *NR3C2* | rs10032216 | T | C | - | 0.67808 | 7648 | 51728 |
| *IGFBP5* | rs13015993 | A | G | - | 0.5131 | 7648 | 51729 |
| *SOX9* | rs9915657 | T | C | - | 0.62625 | 7648 | 51729 |
| *NFIA* | rs334699 | A | G | - | 0.28598 | 7577 | 51653 |
| *FGF7* | rs10519227 | A | T | - | 0.67183 | 7648 | 51725 |
| *PRDM11* | rs17723470 | T | C | + | 0.93846 | 7648 | 51729 |
| *MIR1179* | rs17776563 | A | G | + | 0.60657 | 7537 | 51588 |
| *INSR* | rs4804416 | T | G | + | 0.41633 | 7646 | 51721 |
| *ABO* | rs657152 | A | C | + | 0.041738 | 7647 | 51714 |
| *ITPK1* | rs11624776 | A | C | - | 0.82357 | 7647 | 51703 |
| *NRG1* | rs7825175 | A | G | - | 0.49231 | 7648 | 51727 |
| *MBIP* | rs1537424 | T | C | + | 0.1263 | 7647 | 51724 |
| *SASH1* | rs9497965 | T | C | + | 0.06155 | 7648 | 51725 |
| *GLIS3* | rs1571583 | A | G | - | 0.045503 | 7330 | 50081 |
| **FT4** |  |  |  |  |  |  |  |
| *DIO1* | rs2235544 | A | C | + | 0.39391 | 7648 | 51721 |
| *LHX3* | rs7860634 | A | G | - | 0.24981 | 7464 | 51532 |
| *FOXE1* | rs7045138 | T | C | + | 0.58824 | 7648 | 51715 |
| *AADAT* | rs11726248 | A | G | - | 0.77536 | 6640 | 48210 |
| *LPCAT2* | rs6499766 | A | T | + | 0.94945 | 7646 | 51713 |
| *CAPNS2* | rs7240777 | A | G | - | 0.30645 | 7648 | 51725 |
| Abbreviations: AMD age-related macular degeneration; TSH thyroid-stimulating hormone; FT4 free thyroxine; β = β coefficient found in the original AMD Gene Consortium GWAS; SNP Single Nucleotide Polymorphisms  ^a^ Porcu E et al. PLOS genetics, 2013  ^b^ Fritsche LG et al, Nature genetics, 2013 | | | | | | | |
